# Improved Classification Method Based on the Diverse Density and Sparse Representation Model for a Hyperspectral Image

**DOI:** 10.3390/s19245559

**Published:** 2019-12-16

**Authors:** Na Li, Ruihao Wang, Huijie Zhao, Mingcong Wang, Kewang Deng, Wei Wei

**Affiliations:** 1 School of Instrumentation and Optoelectronic Engineering, Beihang University, Beijing 100191, China; wrh1997@buaa.edu.cn (R.W.); wangmc@buaa.edu.cn (M.W.); dengkewang@buaa.edu.cn (K.D.); 2 Beijing Mechanical and Electrical Engineering Design Institute, Beijing 100854, China; cnkicpb@sina.com

**Keywords:** hyperspectral image classification, small sample size, diverse density, sparse representation

## Abstract

To solve the small sample size (SSS) problem in the classification of hyperspectral image, a novel classification method based on diverse density and sparse representation (NCM_DDSR) is proposed. In the proposed method, the dictionary atoms, which learned from the diverse density model, are used to solve the noise interference problems of spectral features, and an improved matching pursuit model is presented to obtain the sparse coefficients. Airborne hyperspectral data collected by the push-broom hyperspectral imager (PHI) and the airborne visible/infrared imaging spectrometer (AVIRIS) are applied to evaluate the performance of the proposed classification method. Results illuminate that the overall accuracies of the proposed model for classification of PHI and AVIRIS images are up to 91.59% and 92.83% respectively. In addition, the kappa coefficients are up to 0.897 and 0.91.

## 1. Introduction

High spectral resolution makes hyperspectral images easier to distinguish different ground covers, and as a result, the classification of ground covers is one of the important applications of hyperspectral images [1,2,3,4,5]. In the supervised classification, when the number of training samples is determined, the classification accuracy of hyperspectral image will be improved firstly and then be decreased with the increase of the spectral dimension, which is called Hughes phenomenon [6,7]. However, we often fail to provide enough training samples for the classifier of the hyperspectral data with more than 100 bands, and then the inadequate training samples (it is called the small sample size problem, SSS problem) may lead to a decrease of classification accuracy [8,9,10]. There are two common solutions to solve the SSS. One solution is to reduce the dimension of hyperspectral image by using the traditional feature mining technology [11] such as principal component analysis (PCA) [12], and then classify the pixels in the hyperspectral image by using the reduced-dimensional feature vector. However, the use of feature mining sometimes loses some spectral information, which affects the classification accuracy. The other solution is to seek the best compromise between the complexity of the classifier and the learning ability (such as support vector machine, SVM [13,14]). The SVM is based on the structural risk minimization principle. However, the selection of parameters for SVM model such as kernel function and penalty factor often depends on the user’s experience [15,16]. Therefore, sparse representation has become increasingly popular because it does not require much user’s experience.

Recently, sparse representation has shown powerful performance in image classification [17,18,19], restoration [20,21,22], wireless communication [23] and denoising [24]. Sparse representation expresses some signal as a linear combination of a few atoms from a prespecified and over-complete dictionary. There are many ways to construct dictionary groups and set constraints. For example, Zhao et al. introduced low rank constraints into sparse representation to reduce the hyperspectral image noise; Chen et al. proposed an explicit smoothing constraint and spatial-spectral joint sparsity model to classify every pixel; Tu et al. applied correlation coefficient between different pixels and spatial-spectral joint sparsity model for image classification. As Tu said in [17], once the surrounding neighborhood of one pixel includes pixels from different classes, the performance of sparse representation classifier may be seriously decreased. Therefore, how to remove noise from training samples and extract pixels as pure as possible is a crucial issue when the prevalence of mixed pixels in hyperspectral images. Therefore, a novel classification model based on diverse density and sparse representation (NCM_DDSR) is proposed in this paper. Firstly, diversity density (DD) algorithm is presented to obtain the pure atoms of dictionary, which describe the spectral feature. Then each pixel in the hyperspectral images will be decomposed into a linear combination of atoms and the sparse coefficient can be gained by using the constraint-based sparse representation matrix for the improved matching pursuit model. The proposed classifier in this paper can reduce the loss of spectral information and improve the classification accuracy when handling small sample size problem. In addition, there is no need to rely on the user’s experience to select parameters.

## 2. Methodology

### 2.1. Dictionary Learning Based on DD Algorithm

The classification capability based on sparse representation to solve the SSS problem for hyperspectral images is directly affected by the dictionary performance. There are two types of methods to obtain the dictionary. The one is mathematical transformation based method, and the other is the greedy optimization based method. However, it is difficult to obtain an ideal dictionary when there are noises or isomerism problems in hyperspectral images. Therefore, a dictionary learning method based on the DD algorithm is proposed in this paper.

The DD algorithm can process the interfered sample and instance, which is also the basic unit in it. There are two types of instances named positive instance and negative instance. If several instances can describe one interested class of ground cover, they can be considered as a positive training bag for this class of ground cover, otherwise, they will be considered as a negative training bag. For the dictionary learning based on the DD algorithm, the feature vector in the spectral feature space that best represents a class of ground cover is selected as a dictionary atom. Therefore, when the feature vector is sufficiently similar to the positive training bags and sufficiently different from the negative training bags, this feature vector will be selected as the atom describing this interested class.

**Figure 1:**
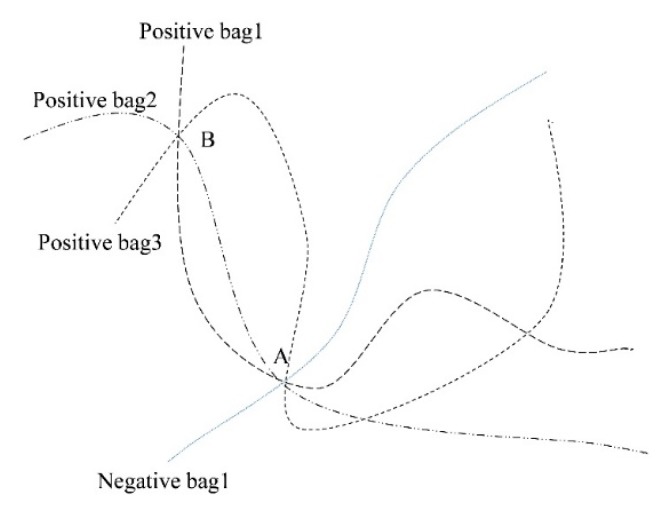
The manifold model of the diversity density (DD) algorithm.

The manifold model of the DD algorithm [25] is shown in Figure 1. It is assumed that each point of the curves represents a feature vector (also known as an instance), all the feature vectors on the four curves are the training samples from the same ground cover, and constitute the spectral feature space of hyperspectral data. The solid line represents the negative bag of this type of ground cover, while the dotted line represents the positive bag of this type of ground cover. A positive training bag contains at least one positive instance and a negative training bag only contains negative instances. The purpose of the DD algorithm is to learn an instance from the positive bags to represent this type of ground cover. The learning strategy of the instance is that the instance must be similar to the positive bags and not to the negative bags. For example, two intersections (A and B) are potential instances, which can be used to represent this type of ground cover because there are three dotted lines passing through the two points. However, there is solid line passes through point A but not go through point B, so point B is more likely to represent this type of ground cover. This model is called a flow model. In fact, there is a function defined to calculate diverse density values in the DD algorithm. In this function, the independent variable is all the feature vectors of the training bags, and the dependent variable is a diverse density value, which can be used to measure the distance between training bags and the feature vectors in spectral feature space. The feature vector, which has the largest DD value, is more likely to be used to represent this type of ground cover.

The description of the DD algorithm for obtaining the atoms of dictionary is as follows. Assume that $B_{i}^{+}$ is the *i*-th positive bag and $B_{ij}^{+}$ is the *j*-th instance in the *i*-th positive bag, while $B_{ijk}^{+}$ is the *k*-th attribute of the j-th instance. Similarly, $B_{i}^{-}$ is the *i*-th negative bag and $B_{ij}^{-}$ is the *j*-th instance in the *i*-th negative bag, and $B_{ijk}^{-}$ is the *k*-th attribute of the *j*-th instance. A multi-dimensional vector in the feature space is marked *x*, and the maximum DD value is represented by $t_{\max}$. $\mathrm{Pr}(x=t|B_{1}^{+},B_{2}^{+},\ldots ,B_{n}^{+},B_{1}^{-},B_{2}^{-},\ldots ,B_{n}^{-})$ is the probability of an instance that belongs to a certain type of ground cover. The learning purpose is to dig $t_{\max}$ out and learn a representative feature vector through maximizing $\mathrm{Pr}(x=t|B_{1}^{+},B_{2}^{+},\ldots ,B_{n}^{+},B_{1}^{-},B_{2}^{-},\ldots ,B_{n}^{-})$. Supposing each instance is subject to independent distribution, according to Bayesian theory:

(1) $$t_{\max}=\arg\max\prod_{i}\mathrm{Pr}(x=t\;|\;B_{i}^{+})\prod_{i}\mathrm{Pr}(x=t\;|\;B_{i}^{-}).$$

Maron and Lozano-Perez used the noisy model to embody (1):

(2) $$\mathrm{Pr}(x=t\;|\;B_{i}^{+})=1-\prod_{j}\left(1-\mathrm{Pr}(x=t\;|\;B_{ij}^{+})\right),$$

(3) $$\mathrm{Pr}(x=t\;|\;B_{i}^{-})=\prod_{j}\left(1-\mathrm{Pr}(x=t\;|\;B_{ij}^{-})\right),$$

where $\mathrm{Pr}(x=t\;|\;B_{ij})=\exp(-{\left\|B_{ij}-x\right\|}^{2})$.

### 2.2. Constraint-Based Sparse Representation

The purpose of the sparse representation (SR) is to represent the spectral feature vectors in the hyperspectral images with a linear combination of as few atoms as possible in the dictionary. Suppose that the pixel of hyperspectral images represented as $x\in R^{k}$, $D=[d^{1},d^{2},\ldots ,d^{i}]\in R^{k\times m}$ (*D* is an atom of the dictionary *D*, $d^{i}$ is the feature vector of a certain ground cover, *m* represents the number of atoms) denotes the dictionary representation learned by training samples. In hyperspectral images, the sparse representation of pixel feature vector *x* based on the dictionary *D* can be shown as follows:

(4) $$\min{\left\|\alpha \right\|}_{0}s.t.\;x=D\alpha .$$

**Figure 2:**
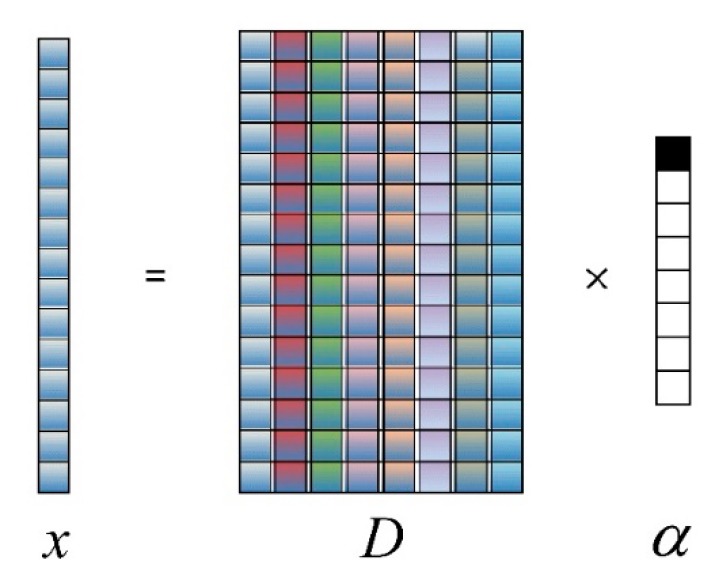
Sparse representation model.

The sparse representation model is shown in Figure 2, where eight different colors in the dictionary *D* represent eight different kinds of ground cover atoms. α represents a sparse coefficient matrix, which the dimension is 1. The coefficient α, which represents a sparse coefficient matrix with 1 dimension, has a threshold. The block shows black when its value is greater than the threshold and the rest shows white. The feature vector x denotes a signal that is sparsely represented. $\alpha \in R^{m}$ is a sparse representation coefficient of pixel feature vector *x* in hyperspectral images based on the dictionary *D*. ${\left\|\cdot \right\|}^{2}$ denotes *l*_0_-norm, which is defined as the number of non-zero elements in the vector α. To the mixed pixel effect of hyperspectral image, a sparse representation model based on relaxation constraint is proposed. The sparse representation equality constraint can be relaxed to the following inequality by adding a relaxation factor ξ:

(5) $$\min{\left\|\alpha \right\|}_{0}s.t.\;\left\|x-D\alpha \right\|\leq \xi .$$

In solving the *l*_0_-norm optimization problem, an improved matching pursuit algorithm is introduced to compute the sparse coefficient, which is non-negative and added up to 1.

The pixel feature vector of the hyperspectral images *x* is projected to an atom of the dictionary $\alpha \in R^{m}$, and calculate the remainder vector of pixel feature vector *Rx* based on the selected dictionary atom. The description is shown as follows:

(6) $$x=<x,d^{i}>d^{i}+Rx.$$

Orthogonalized by *Rx* and $d^{i}$, we can obtain Formula (7):

(7) $${\left\|x\right\|}^{2}=|<x,d^{i}>{|}^{2}+{\left\|Rx\right\|}^{2}.$$

To make ‖Rx‖ as small as possible, the selected $d^{i}\in D$ needs to make $<x,d^{i}>$ as larger as possible. Considering the physical meaning of hyperspectral remote sensing mixed pixels, the atoms coefficient in the sparse representation model should be greater than zero, which is equal to the values $<x,d^{i}>$ of the reserved atoms are positive and the values $<x,d^{i}>$ of the ignored atoms are negative.

The residual is decomposed in the matching pursuit algorithm. Suppose *m*-th residual in $R^{0}x=x$ has been obtained, it is necessary to select $d^{j}\in D$ in the next step or iteration and it should be satisfied:

(8) $$<R^{m}x,d^{j}>{|}^{2}\geq \sup<R^{m}x,d^{j}>.$$

Projected $R^{m}x$ to $d^{j}$:

(9) $$R^{m}x=<R^{m}x,d^{j}>+R^{m+1}x.$$

All components of x from m=0 to m=M−1 is summed, and then x can be expressed as:

(10) $$x=\sum_{m=0}^{M-1}<R^{m}x,d^{m}>d^{m}+R^{M}x.$$

In (10), M is the number of iterations. The following (11) is obtained by orthogonal transformation.

(11) $${\left\|x\right\|}^{2}={\sum_{m=0}^{M-1}\left|<R^{m}f,d^{m}>\right|}^{2}+{\left\|R^{M}x\right\|}^{2}.$$

The pixel’s sparse coefficient vector can be acquired with arranging all the coefficients of the atoms by the order in dictionary. The sparse coefficients can be obtained by calculating all the pending pixels in the hyperspectral remote sensing images. Finally, the sparse coefficients are applied to classify different ground covers.

### 2.3. NCM_DDSR Algorithm

This article introduces a hyperspectral image classifier that combines DD and SR. The proposed NCM_DDSR is composed of two components (DD and SR). The major steps of the proposed method can be concluded in Algorithm 1.


**Algorithm 1 Novel classification method based on diverse density and sparse representation**
**Inputs:** Positive bag $B_{i}^{+}$ and negative bag $B_{i}^{-}$ for hyperspectral images; *Pi* is the number of the *i*-th training sample bags; *i* = {1, …, *N*} is the number of bags; *j* = {1, …, *Pi*} is the number of instances; *b* = {1, …, *P*} is the number of training samples; *x* is a pixel to be sparse represented; $d^{i}$ is an atom in the dictionary *D*; *Rx* is the current residual *Step 1* Diverse Density. **for**
*b* = 1, …, *P* **for**
*j* = 1, …, *N* **for**
*i* = 1, …, *Pi* Judge the sample package based on (2), (3) **end for** Calculate the *t*_max_ based on (1) **end for** Compare to find the largest *t*_max_ **end for** *Step 2* Use the Diverse Density algorithm to obtain labels and feature vectors to build a dictionary *D*. *Step 3* Sparse representation. **do** {Select $d^{i}$ which maximizes |$<x,d^{i}>$|} **while** ($<x,d^{i}>$ is positive) **do** {Calculate the atomic coefficients based on (11)} **while** (The sum of the atomic coefficients is equal to 1) *Step 4* Determine the class label of every test pixel. **Outputs:** The classification map.

First, use the DD algorithm to obtain the dictionary atom corresponding to each category. Second, normalize each dictionary atoms generated in Step 1 and combine the dictionary atoms into a dictionary. Third, sparse coefficient matrix is calculated by RS algorithm. In detail, when the test data *x* is normalized, the sparse coefficient corresponding to the *i*-th dictionary atom is $<x,d^{i}>$. After several iterations, a sparse coefficient matrix that satisfies the discrimination conditions can be obtained. Fourth, the class label of each pixel is determined based on the decision function. In our proposed method, the classification decision is sparse coefficient matrix and least squares classification. When an element in the sparse coefficient vector is greater than 0.5, the pixel is directly classified as the class corresponding to the element. When the value of all elements is less than 0.5, the class of this pixel is determined using the minimum Euclidean distance. Euclidean distance $r^{k}(x)$ is calculated as (12), where $\alpha^{k}$ is the sparse matrix of the *k*-th class.

(12) $$r^{k}(x)={\left\|x-D^{k}\alpha^{k}\right\|}_{2}.$$

By comparing the Euclidean distances of all classes, the class of *x* can be determined based on (13). *k* is the number of categories.

(13) $$Class(x)=\arg\underset{k=1,\ldots K}{\min}r^{k}(x).$$

## 3. Experiment

**Figure 3:**
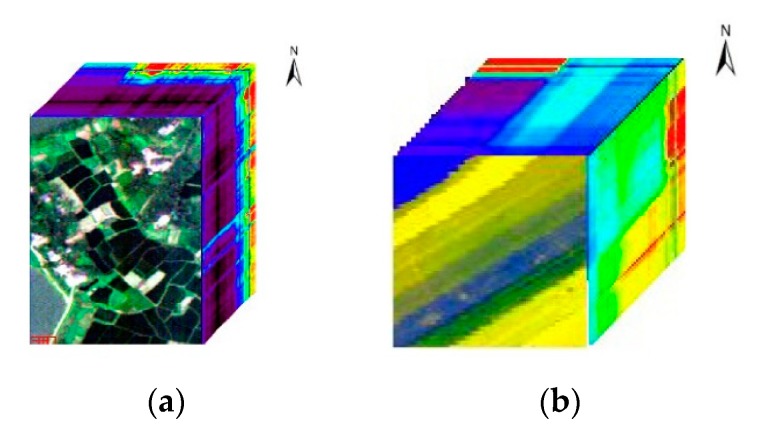
Data cubes of experimental images. (**a**) Data cube of push-broom hyperspectral imager (PHI) and (**b**) data cube of airborne visible/infrared imaging spectrometer (AVIRIS).

**Figure 4:**
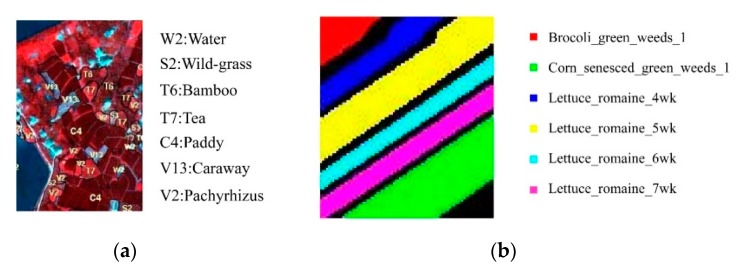
Ground truth of research area: (**a**) ground truth of PHI data and (**b**) ground truth of AVIRIS data.

In order to evaluate the performance of NCM_DDSR solving SSS problem, the data collected by push-broom hyperspectral imager (PHI) in the Fanglu tea plantation area, Jiangsu province and the airborne visible/infrared imaging spectrometer (AVIRIS) in Salinas, American are used. The PHI data with a size of 200 pixels × 150 pixels whose spectral range is from 455.8 to 804.4 nm with 6 nm spectral resolution has 65 spectral channels. There are seven classes of ground cover in PHI data, namely paddy, caraway, wild-grass, *Pachyrhizus*, tea, bamboo, and water. The AVIRIS data whose spectral range is from 370.49 to 2507.59 nm with 9.7 nm spectral resolution is 86 pixels × 83 pixels and has 166 spectral channels apart from water vapor absorption bands. There are six classes of ground cover, which is brocoli_green_weeds_1, corn_senesced_green_weeds, lettuce_romaine_4wk, lettuce_romaine_5wk, lettuce_romaine_6wk and lettuce_romaine_7wkv respectively. The hyperspectral data cube of the PHI data and the AVIRIS data is shown in Figure 3a,b respectively. Figure 4 shows the corresponding ground truth.

To evaluate the performance of classification methods in solving SSS problem, some classification experiments are carried out under the small sample condition. Since the SSS problem is relative to the spectral dimension of the hyperspectral remote sensing images, it can be considered as a small sample when the number of training samples is less than one times the number of bands. The number of training samples we selected was 0.5 times of bands. The proposed NCM_DDSR classifier (sparse representation classifier based on multi-instance learning), minimum distance classifier based on principal component analysis (PCA_MinD) and SVM classifiers [25] were applied to analyze the classification capability.

### 3.1. PHI Image

**Figure 5:**
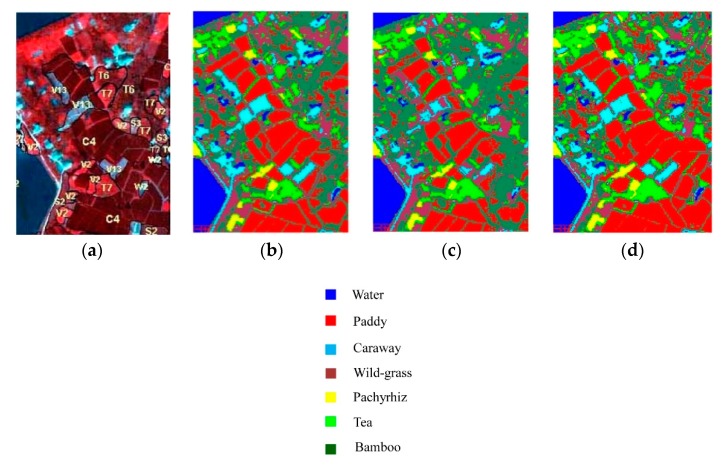
Classification results of PHI data with different methods (the ratio of the number of samples to the number of bands is 0.5): (**a**) ground truth; (**b**) PCA_MinD; (**c**) SVM and (**d**) NCM_DDSR.

The classification results of Changzhou Fanglu Tea plantation area based on PCA_MinD, SVM and NCM_DDSR classifier are shown in Table 1, where the number of samples (a total of 28 training samples, four samples per ground cover) was 0.5 times the bands. The ground truth is shown in Figure 5a and the classification results of the three classifiers are shown in Figure 5b–d. From Figure 5 and Table 1, it was illuminated that when the number of training samples was 0.5 times the bands, NCM_DDSR had the best classification performance, far exceeding the other methods. Moreover, from the result of overall and each ground cover accuracy, NCM_DDSR achieves high classification accuracy in the case of small samples (reaching 91.59%) and had the highest classification consistency with a Kappa coefficient up to 0.90.

### 3.2. AVIRIS Image

**Figure 6:**
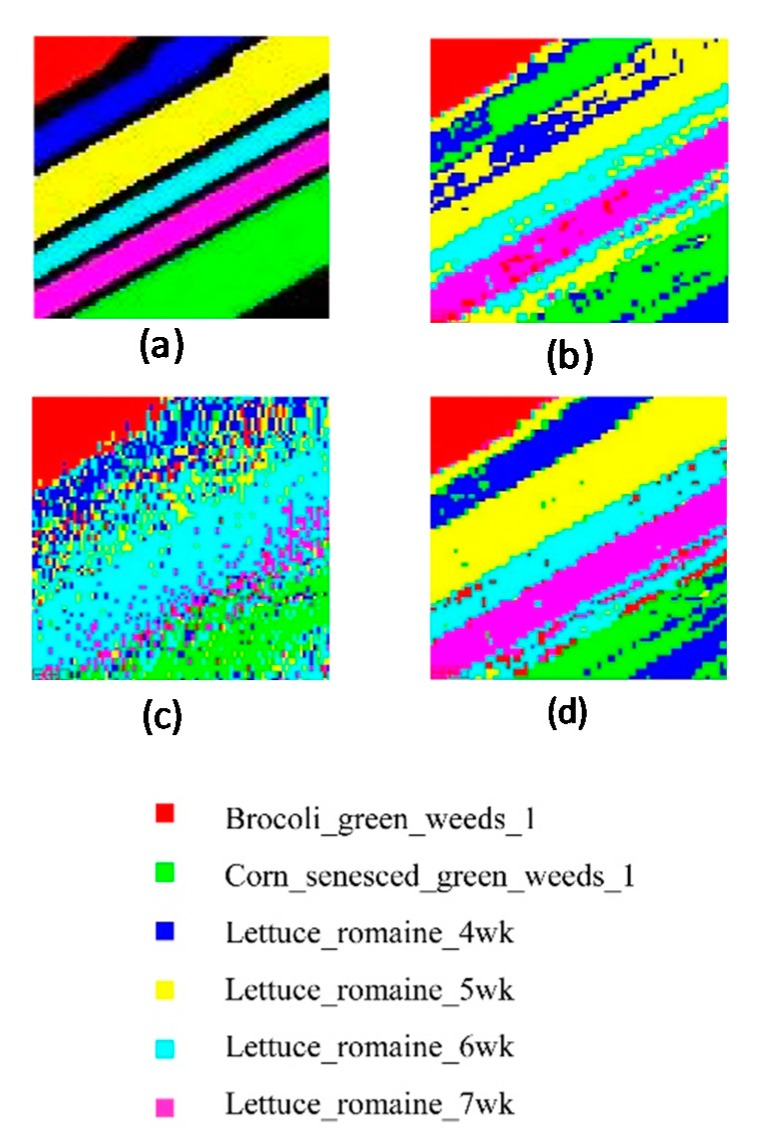
Classification results of Salinas_A (the ratio of the number of samples to the number of bands is 0.5). (**a**) Ground truth. (**b**) PCA_MinD. (**c**) SVM. (**d**) NCM_DDSR.

To further evaluate the classification capability of NCM_DDSR in the case of small samples, the Salinas_A hyperspectral data collected by AVIRIS was used. The number of training samples (a total of 84 training samples, 14 samples per ground cover) was 0.5 times the bands. Figure 6 shows the classification results including the ground truth (Figure 6a and the classified results of the PCA_MinD, SVM and NCM_DDSR classifiers (Figure 6b–d respectively)). As the overall classification accuracy and Kappa coefficients of the above three classifiers shown in Table 2, the classification accuracy obtained by our study was significantly higher than the other two methods even up to 92.83%. However, in Figure 6, the classification result for the class of Lettuce_romaine_7wk using the SVM method is seriously problematic and cannot meet the application requirements, just as the classification result for the Lettuce_romaine_4wk acquired by PCA_MinD also makes a serious error.

## 4. Discussion

From the above-mentioned classification results, our study was clearly better than PCA_MinD and SVM methods in the case of SSS. To discuss the effects of the classification performance of the proposed method with different training sample numbers, we used AVIRIS and PHI data and gave the overall accuracy, Kappa coefficient and the classified results, respectively.

### 4.1. PHI Image

The classification results of the hyperspectral data in Changzhou Fanglu Tea plantation area using the PCA_MinD, SVM and NCM_DDSR classifier are shown in Table 3, when the ratio of the number of samples to the number of bands was 1.

From Table 3, the classification accuracy results of the proposed method (NCM_DDSR) in this paper were the best when the number of training samples is approximately equal to the number of image bands. The classification results of PCA_MinD and SVM methods were similar and less than NCM_DDSR. Furthermore, the overall accuracy of NCM_DDSR classifier could reach higher classification accuracy up to 91.88% and its Kappa coefficient was 0.90 under the small sample condition, which had a higher classification consistency. On the contrary the overall accuracy of the PCA_MinD classifier was 87.78% and the Kappa coefficient was 0.85. Compared with the case where the number of samples was 0.5 times the number of bands, the overall accuracy and Kappa coefficient of the PCA_MinD method were slightly increased. For example, the overall accuracy of SVM classifier was 88.14% and the Kappa coefficient was 0.858, the classification result was greatly improved when the number of samples was 0.5 times the number of bands.

Table 4 shows the classification results of three classifiers PCA_MinD, SVM and NCM_DDSR when the ratio of the number of samples to the number of bands was 1.5. From Table 4, the classification performance of NCM_DDSR was optimum when the number of training samples was 1.5 times the number of bands. The classification accuracy of the three classifiers was approximately equal. The NCM_DDSR classifier could obtain the overall accuracy of 92% and its Kappa coefficient was 0.9. It had a very high classification agreement.

Table 5 shows the statistics of the classification capability of the three classifiers with the number of training samples. From Table 5, the classification result of the proposed method did not decrease substantially as the number of samples decreased and its overall accuracy reached up to 90%, and it had obvious superiority in the case of a small number of training samples. The classification results of PCA_MinD and SVM method decreased significantly with the decrease in the number of training samples, especially the SVM method. When there were enough training samples, the classification results of the three classifiers tended to be consistent. Therefore, the proposed NCM_DDSR method had significant advantages for solving the small sample problems compared with PCA_MinD and SVM methods.

### 4.2. AVIRIS Image

Table 6 shows the classification performance statistics of the three classifiers when the training samples were 0.5 times, one times (a total of 168 training samples, 28 samples per class) and 1.5 times (a total of 252 training samples, 42 samples per class) the number of bands for Salinas_A hyperspectral images, respectively.

It can be seen from the classification results that our method obtained better classification results, and the classification accuracy was higher than 90%. As the number of training samples decreases, the classification accuracy of PCA_MinD and SVM classifier were both decreased, especially the SVM method (its Kappa coefficient was merely 0.52 when the training samples were 0.5 times the number of bands). Therefore, the proposed method in this paper was competitive in the case of small samples.

## 5. Conclusions

The main contribution of this paper was to explore how to obtain higher classification accuracy in the face of SSS problems. The NCM_DDSR method based on diverse density and sparse representation was proposed in this paper. In our proposed method, the diversity density (DD) algorithm was introduced to obtain the dictionary atoms that described the spectral feature, and an improved sparse representation model based on the relaxation constraint was proposed. The sparse coefficient vector could be acquired with arranging all the coefficients of the atoms by the order in the dictionary for matching pursuit model. The NCM_DDSR method was compared with several classical hyperspectral image classification methods, such as PCA_MinD and SVM method, by using the PHI data and AVIRIS data. The classification results illuminated that the classifier based on the NCM_DDSR method performed better than that based on PCA_MinD and the SVM method. Specifically, the overall accuracy and kappa coefficient of the proposed classifier were much higher than the SVM and PCA_MinD classifiers in the case of small training samples. The results show that the NCM_DDSR classifier could handle the SSS problem effectively in the experiments with different data and different training samples.

## Figures and Tables

**Table 1:** Classified results of Fanglu Tea plantation (the ratio of the number of samples to the number of bands is 0.5).

Category	PCA_MinD	SVM	NCM_DDSR
Water (%)	92.83 ± 1.76	85.00 ± 1.73	89.33 ± 4.54
Paddy (%)	75.00 ± 0.00	58.50 ± 0.00	99.50 ± 0.00
Caraway (%)	100.0 ± 0.00	86.67 ± 23.0	100.0 ± 0.00
Wild-grass (%)	89.67 ± 4.62	94.67 ± 1.15	90.67 ± 1.53
Pachyrhizus (%)	80.00 ± 1.73	77.67 ± 1.15	83.00 ± 5.20
Tea (%)	97.67 ± 1.53	98.33 ± 2.89	100.0 ± 0.00
Bamboo (%)	75.33 ± 8.14	87.67 ± 8.50	73.00 ± 6.08
Overall accuracy (%)	86.48 ± 1.67	81.33 ± 1.86	91.59 ± 1.77
Kappa coefficient	0.840 ± 0.02	0.779 ± 0.02	0.897 ± 0.02

**Table 2:** Classification results of Salinas_A (the ratio of the number of samples to the number of bands is 0.5).

Method	Overall Accuracy (%)	Kappa Coefficient
PCA_MinD	68.16 ± 7.19	0.61 ± 0.09
SVM	60.50 ± 1.48	0.52 ± 0.02
NCM_DDSR	92.83 ± 1.02	0.91 ± 0.01

**Table 3:** Classified results of Fanglu Tea plantation (the ratio of the number of samples to the number of bands is 1).

Category	PCA_MinD	SVM	NCM_DDSR
Water (%)	93.67 ± 0.76	84.83 ± 1.44	95.1 ± 0.29
Paddy (%)	83.33 ± 4.04	81.50 ± 0.50	99.33 ± 0.58
Caraway (%)	100.0 ± 0.00	98.67 ± 2.31	100.0 ± 0.00
Wild-grass (%)	89.67 ± 4.73	96.33 ± 1.15	85.33 ± 6.35
Pachyrhizus (%)	82.00 ± 2.65	85.00 ± 5.29	97.00 ± 0.00
Tea (%)	95.33 ± 1.53	94.67 ± 4.16	94.33 ± 4.04
Bamboo (%)	69.00 ± 5.20	86.00 ± 8.19	61.33 ± 13.2
Overall accuracy (%)	87.78 ± 1.25	88.14 ± 0.74	91.88 ± 1.54
Kappa coefficient	0.850 ± 0.02	0.858 ± 0.01	0.900 ± 0.02

**Table 4:** Classified results of Fanglu tea plantation (the ratio of the number of samples to the number of bands is 1.5).

Category	PCA_MinD	SVM	NCM_DDSR
Water (%)	93.00 ± 0.00	84.00 ± 0.00	93.67 ± 2.36
Paddy (%)	91.50 ± 6.93	98.17 ± 1.15	98.50 ± 0.87
Caraway (%)	100.0 ± 0.00	100.0 ± 0.00	100.0 ± 0.00
Wild-grass (%)	87.00 ± 1.00	96.33 ± 0.58	86.67 ± 5.86
Pachyrhizus (%)	84.33 ± 2.08	87.33 ± 3.79	89.67 ± 6.03
Tea (%)	95.33 ± 2.31	98.67 ± 1.15	98.67 ± 1.15
Bamboo (%)	76.33 ± 1.53	85.33 ± 6.66	68.67 ± 6.43
Overall accuracy (%)	90.22 ± 1.75	92.44 ± 0.70	92.00 ± 1.61
Kappa coefficient	0.880 ± 0.02	0.903 ± 0.01	0.902 ± 0.02

**Table 5:** Overall accuracy and Kappa coefficient of three classifiers under different number of training samples for PHI data.

Ratio of Sample to Band Number	0.5		1		1.5	
Accuracy Evaluation Index	Overall Accuracy	Κ Coefficient	Overall Accuracy	Κ Coefficient	Overall Accuracy	Κ Coefficient
PCA_MinD	86.48%	0.840	87.78%	0.850	90.22%	0.880
SVM	81.33%	0.779	88.14%	0.858	92.44%	0.903
NCM_DDSR	91.59%	0.897	91.88%	0.900	92%	0.902

**Table 6:** Overall accuracy and Kappa coefficient of three classifiers under different number of training samples for AVIRIS data.

Ratio of Sample to Band Number	0.5		1		1.5	
Accuracy Evaluation Index	Overall Accuracy	Κ Coefficient	Overall Accuracy	Κ Coefficient	Overall Accuracy	Κ Coefficient
PCA_MinD	68.16%	0.61	84.05%	0.81	87.88%	0.85
SVM	60.50%	0.52	72.11%	0.66	79.94%	0.75
NCM_DDSR	92.83%	0.91	92.94%	0.91	92.50%	0.90
